# Supplementary material for: Impact of the Oral Administration of Polystyrene Microplastics on Hepatic Lipid, Glucose, and Amino Acid Metabolism in C57BL/6Korl and C57BL/6-Lepem1hwl/Korl Mice
Source: Int J Mol Sci. 2024 May 2;25(9):4964. doi: 10.3390/ijms25094964 (PMC11084201; doi:10.3390/ijms25094964)
Supplement: Supplementary file 1 [file ijms-25-04964-s001.zip › ijms-2928278-supplementary.pdf]

Supplementary Table S1. Primer sequences for PCR and RT-qPCR analyses

| Primer name | Sequence (from 5' to 3') | Product size (bp) |
|---|---|---|
| Leptin | | |
| Forward | TCC CAG GGA GGA AAA TGT GCT | 219 |
| Reverse | TGA CAT GTT TCT CAG ACT CTG GTT | |
| PPARγ | | |
| Forward | GAG TTC ATG CTT GTG AAG GAT GCA AGG | 80 |
| Reverse | CAT ACT CTG TGA TCT CTT GCA CG | |
| C/EBPα | | |
| Forward | GTG GAC AAG AAC AGC AAC GAG TAC | 71 |
| Reverse | GGA ATC TCC TAG TCC TGG CTT GC | |
| FAS | | |
| Forward | GAT CCT GGA ACG AGA ACA CGA TCT GG | 128 |
| Reverse | AGA CTG TGG AAC ACG GTG GTG GAA CC | |
| aP2 | | |
| Forward | GAA CCT GGA AGC TTG TCT CCA GTG GAT | 72 |
| Reverse | GCT CTT CAC CTT CCT GTC GTC TGC | |
| GLUT4 | | |
| Forward | GCT TTG TGG CCT TCT TTG AGA | 67 |
| Reverse | CTG AAG AGC TCT GCC ACA ATG | |
| Insulin receptor | | |
| Forward | AAT GGG ACC ACT GTA TGC ATC TT | 84 |
| Reverse | TCG TCC GGC ACG TAC ACA | |
| PYGL | | |
| Forward | CCA GAA TAA AAC CAA CGG GAT TA | 88 |
| Reverse | TCC AAT TTT CTC CGC TAT CAA GTC | |
| Phosphoglucomutase | | |
| Forward | GGA GCT ACT CTC TGG TCC AAA CA | 76 |
| Reverse | CTT TAC GTA CGG TCC CAC AAC TC | |
| G6PC3 | | |
| Forward | CCA GTT CCC TTC TTC TTG TGA GA | 71 |
| Reverse | GAG CTG CGC CTG TGA TCA | |
| β-actin | | |
| Forward | TGG AAT CCT GTG GCA TCC ATG AAA C | 349 |
| Reverse | TAA AAC GCA GCT CAG TAA CAG TCC G | |

Abbreviations: PPARγ; peroxisome-proliferator activator receptor γ, C/EBPα; CCAAT/enhancer binding protein α, FAS; fatty acid synthase, aP2; adipocyte protein 2, GLUT4; Glu transporter type 4, PYGL; glycogen phosphorylase L, G6PC3; Glu-6-phosphatase catalytic subunit 3.

Supplementary Table S2. Definition of the NAFLD score

| Criteria | Definition | Score |
|---|---|---|
| Steatosis | | |
| Grade | < 5% | 0 |
| | 5% ~ 33% | 1 |
| | > 33%~66% | 2 |
| | > 66% | 3 |
| Location | Zone 3 | 0 |
| | Zone 1 | 1 |
| | Azonal | 2 |
| | Panacinar | 3 |
| Microvesicular steatosis | Absent | 0 |
| | Present | 1 |
| Inflammation | | |
| Lobular inflammation | No foci | 0 |
| | < 2 foci per 200× field | 1 |
| | 2-4 foci per 200× field | 2 |
| | > 4 foci per 200× field | 3 |
| Microgranulomas | Absent | 0 |
| | Present | 1 |
| Large lipogranulomas | Absent | 0 |
| | Present | 1 |
| Portal inflammation | None to minimal | 0 |
| | Greater than minimal | 1 |
| Ballooning | None | 0 |
| | Few ballooned cells | 1 |
| | Many/prominent ballooned cells | 2 |

Abbreviation: NAFLD; Non-alcoholic fatty liver disease.

Supplementary Table S3. List of antibodies for Western blot assay

| Antibody name | Sequence (from 5' to 3') | Dilution ratio |
| --- | --- | --- |
| anti-ATGL antibody | Cell Signalling Technology Inc., Danvers, MA, USA | 1:1,000 |
| anti-HSL antibody | Cell Signalling Technology Inc., Danvers, MA, USA | 1:1,000 |
| anti-p-HSL antibody | Invitrogen, Carlsbad, CA, USA | 1:1,000 |
| anti-perilipin antibody | Cell Signalling Technology Inc., Danvers, MA, USA | 1:1,000 |
| anti-p-perilipin antibody | Cell Signalling Technology Inc., Danvers, MA, USA | 1:1,000 |
| anti-AMPK antibody | Cell Signalling Technology Inc., Danvers, MA, USA | 1:1,000 |
| anti-p-AMPK antibody | Cell Signalling Technology Inc., Danvers, MA, USA | 1:1,000 |
| anti-GLUT4 antibody | Invitrogen, Carlsbad, CA, USA | 1:1,000 |
| anti-insulin receptor β | Cell Signalling Technology Inc., Danvers, MA, USA | 1:1,000 |
| anti-IRS-1 antibody | Cell Signalling Technology Inc., Danvers, MA, USA | 1:1,000 |
| anti-p-IRS-1 antibody | Cell Signalling Technology Inc., Danvers, MA, USA | 1:1,000 |
| anti-PI3K antibody | Cell Signalling Technology Inc., Danvers, MA, USA | 1:1,000 |
| anti-p-PI3K antibody | Cell Signalling Technology Inc., Danvers, MA, USA | 1:1,000 |
| anti-Akt antibody | Cell Signalling Technology Inc., Danvers, MA, USA | 1:1,000 |
| anti-p-Akt antibody | Cell Signalling Technology Inc., Danvers, MA, USA | 1:1,000 |
| anti-β-actin antibody | Cell Signalling Technology Inc., Danvers, MA, USA | 1:1,000 |

Abbreviations: ATGL; Adipose triglyceride lipase, HSL; hormone-sensitive lipase, AMPK; 5' AMP-activated protein kinase, GLUT4; Glu transporter type 4, IRS-1; insulin receptor substrate 1, PI3K; phosphatidylinositol 3-kinase, Akt; protein kinase B.
